# Elucidating Axonal Injuries Through Molecular Modelling of Myelin Sheaths and Nodes of Ranvier

**DOI:** 10.3389/fmolb.2021.669897

**Published:** 2021-06-23

**Authors:** Marzieh Saeedimasine, Annaclaudia Montanino, Svein Kleiven, Alessandra Villa

**Affiliations:** ^1^ Department of Biosciences and Nutrition, Karolinska Institutet, Huddinge, Sweden; ^2^ Division of Neuronic Engineering, KTH-Royal Institute of Technology, Stockholm, Sweden; ^3^ PDC-Center for High Performance Computing, KTH-Royal Institute of Technology, Stockholm, Sweden

**Keywords:** coarse grained model, molecular dynamics simulations (MD simulations), brain injury, axonal membrane, mechanoporation

## Abstract

Around half of the traumatic brain injuries are thought to be axonal damage. Disruption of the cellular membranes, or alternatively cytoskeletal damage has been suggested as possible injury trigger. Here, we have used molecular models to have a better insight on the structural and mechanical properties of axon sub-cellular components. We modelled myelin sheath and node of Ranvier as lipid bilayers at a coarse grained level. We built ex-novo a model for the myelin. Lipid composition and lipid saturation were based on the available experimental data. The model contains 17 different types of lipids, distributed asymmetrically between two leaflets. Molecular dynamics simulations were performed to characterize the myelin and node-of-Ranvier bilayers at equilibrium and under deformation and compared to previous axolemma simulations. We found that the myelin bilayer has a slightly higher area compressibility modulus and higher rupture strain than node of Ranvier. Compared to the axolemma in unmyelinated axon, mechanoporation occurs at 50% higher strain in the myelin and at 23% lower strain in the node of Ranvier in myelinated axon. Combining the results with finite element simulations of the axon, we hypothesizes that myelin does not rupture at the thresholds proposed in the literature for axonal injury while rupture may occur at the node of Ranvier. The findings contribute to increases our knowledge of axonal sub-cellular components and help to understand better the mechanism behind axonal brain injury.

## 1 Introduction

Traumatic brain injury (TBI) (Menon et al., 2010) is an injury to the brain caused by an external force. Causes include falls, vehicle collisions and violence. One of the most common consequence of a TBI is diffuse axonal injury, a multifocal damage to white matter (Johnson et al., 2013). Such an injury is invisible to conventional brain imaging, and can only be histologically diagnosed and one of its hallmarks is the presence of axonal swellings (Hill et al., 2016).

Axons are long projection of the nerve cell surrounded by a membrane, called axolemma. Axolemma separates the interior of the axon from the outside environments. In the nervous system, axons may be myelinated, or unmyelinated. When the axon is myelinated, a extra multilamellar membrane, called myelin sheath, insulates segments of axon. The sheath consists of repeating units of double bilayers separated by 3–4 nm-thick aqueous layers that alternate between the cytoplasmic and extracellular faces of membranes (Inouye and Kirschner, 1988a). Dehydrated myelin is unusual in that it is composed of 75–80% lipid and 20–25% protein by weight, compared with around 50% of lipids in most other cell membranes (Williams et al., 1993). Multiple lipids make up the myelin sheath, and each sheath contributes to the structure, adhesive stability, and possibly the pathogenesis of the myelin membrane. The asymmetric distribution of lipid composition on the cytoplasmic and extracellular faces likely also plays an important role (Inouye and Kirschner, 1988b). Axolemma and myelin compositions differ both in lipid type and degree of saturation. Table 1 summarizes the available experimental data on lipid composition for axolemma and myelin. The most striking feature of myelin lipid composition is the enrichment in glycolipids together with long-chain fatty acids (DeVries et al., 1981).

**TABLE 1:** Experimental and modelled lipid composition in weight fraction for different types of membrane.

Lipid type Membrane	PC	PE	SM	PS	Glycolipid	CHOL	Other
Myelin (exp)^a^	0.11	0.17	0.06	0.06	0.28	0.28	0.04
	(0.01)	(0.01)	(0.03)	(0.01)	(0.03)	(0.01)	(0.02)
Myelin model	0.12	0.22	0.03	0.09	0.23	0.29	0.02
Axolemma (exp)^b^	0.20	0.19	0.08	0.06	0.20	0.24	0.10
	(0.09)	(0.07)	(0.05)	(0.03)	(0.05)	(0.03)	(0.07)
Plasma model^c^	0.30	0.17	0.17	0.06	0.05	0.17	0.08

For composition observed experimentally the average values is reported together with the maximum deviations from the mean values (in parentheses).

a data for the central nervous system of human, bovine and rat from Rasband and Macklin (2012) and references inside.

b data from central and peripheral nervous system from Camejo et al. (1969); Zambrano et al. (1971); DeVries et al. (1981); DeVries et al. (1999).

c plasma membrane model of Ingólfsson et al. (2014) was used to describe the axolemma.

Short unmyelinated segments, nodes of Ranvier, occur periodically between segments of the myelin sheath in myelineted axons. In nodes of Ranvier, the axolemma is directly exposed to the extracellular space. The nodes are uninsulated and highly enriched in ion channels (Westenbroek et al., 1989; Catterall et al., 2005), allowing them to participate in the exchange of ions required to regenerate the action potential. One of the voltage-gated ion channels, embedded in myelinated axons, is sodium channel protein type subunit alpha, Nav1.1 (Duflocq et al., 2008). In axonal membrane, the density of sodium channels varies between 5 and 3,000 channels/μm^2^: lower densities are observed in unmyelinated axons, while higher densities are found in the nodal portions of myelinated axons (Wann, 1993; Hu and Jonas, 2014). Interestingly, sodium channels have also been proposed to influence the injury response (Iwata, 2004).

Experimental observations have so far led to the formulation of two main theories regarding the cellular primary injury mechanism. Disruption of the axolemma (Pettus and Povlishock, 1996; LaPlaca and Thibault, 1997; Fitzpatrick et al., 1998), or alternatively cytoskeletal damage (Tang-Schomer et al., 2012) has been suggested mainly as injury trigger. However, using a purely mechanical approach we discarded microtubule damage as injury trigger and revealed instead high level of strains on the axonal membrane (Montanino and Kleiven, 2018). To further investigate the molecular level effects of such strains, we bridged the finite element model of the axon with a molecular-based membrane model (Montanino et al., 2020). Despite the approximation of the models, we showed that in a typical injury scenario, the axonal cortex sustains deformations large enough to entail pore formation in the adjoining lipid bilayer. The observed axonal deformation at which poration occurs (10–12%) agrees well with the thresholds obtained both with *in-vitro* (Hemphill et al., 2011; Nakadate et al., 2017) and *in-vivo*/*ex-vivo* (Bain and Meaney, 2000; Singh et al., 2015) stretch injury experiments and allows us to provide quantitative evidences that do not exclude pore formation in the membrane as a result of trauma.

When investigating the progression of axonal injury in the white matter, several studies have observed impairments at the myelin level in the form of delamination of the myelin lamellae (Maxwell, 2013) and general loss of myelin, or demyelination, (Mierzwa et al., 2015; Mu et al., 2019). While white matter degeneration is undoubtedly a distinctive feature of traumatic brain injury, the causal relationships between myelin disruption and the multitude of events associated with axonal damage is still unclear. Computational models of the axon can potentially clarify the damage-causality chain, provided that a mechanical description of myelin is obtained.

Here, we aim to describe membrane-component of myelinated axon at molecular level and to elucidate if and where membrane rupture can occur as a result of an injury. To achieve this, we have used molecular-based model for myelin sheath and for node of Ranvier in line with experimental composition (Table 1) and a coarse grained (CG) description. For myelin, we have built ex-novo the model based on experimental lipid composition and saturation from central nervous system (O’Brien and Rouser, 1964; Manzoli et al., 1970; Inouye and Kirschner, 1988b; Bosio et al., 1998). For the node of Ranvier, we have used an already available plasma membrane model (Ingólfsson et al., 2014), since too few experimental data on the axolemma composition for central nervous system are available. Molecular dynamics (MD) simulations have been used to characterize the molecular system at equilibrium and under deformation. Finally the deformation results from molecular simulations are combined with the axonal deformation obtained using an axonal finite element (FE) model (Montanino et al., 2020).

## 2 Methods

### 2.1 Molecular Model

#### 2.1.1 Model for Myelin Sheath

The myelin is described as lipid bilayer. The membrane model contains 17 different types of lipids, distributed asymmetrically between two leaflets, labelled as extracellular and cytoplasmic leaflet. The extracellular leaflet is characterized by having phosphatidylcholine (PC) (7%), phosphatidylethanolamine (PE) (8%), cholesterol (CHOL) (43%), cerebroside (39%), sphingomyelin (SM) (2%), phosphatidylserine (PS) (1%) and other anionic lipids, while the cytoplasmic leaflet contains PC (11%), PE (27%), CHOL (44%), SM (3%), PS (12%) and other anionic lipids. Note that the reported values are in mol%. Supplementary Table S1 lists all the lipid types used and their disposition in the leaflets together with the level of saturation of fatty acids. The membrane model was built accounting the available experimental data: we use the values derived by Inouye and Kirschner (Inouye and Kirschner, 1988b) for myelin in central neurons system to define the lipid disposition in the extracellular and cytoplasmic leaflet; the work of Manzoli and coworkers (Manzoli et al., 1970) to define the saturation of the lipid tails for phospholipids, while the saturation values for cerebroside have obtained from O’Brien and Rouser (1964) and Bosio et al. (1998).

#### 2.1.2 Model for Node of Ranvier

To describe the region of Node of Ranvier, we use a lipid bilayer embedded with ion channels. A channel concentration of 3,086 channels/was used to describe the node. The mammalian plasma membrane model designed by Ingólfsson et al. (2014) and deposited on MARTINI webpage (http://www.cgmartini.nl/) was used to describe the membrane. The model contains 63 different types of lipids distributed asymmetrically between two leaflets. The extracellular leaflet has a higher level of tails saturation and contains PC (36%), PE (6%), CHOL (31%), SM (19%), glycolipids (6%), and other lipids (2%). The cytoplasmic leaflet, which has a higher level of polyunsaturation, contains PC (17%), PE (25%), CHOL (29%), SM (9%), PS (11%), anionic phosphatidylinositol (PI) (2%), and other lipids (7%).

As model for the protein we used the *Homo sapiens* Nav1.1. The three-dimensional (3D) structure of Nav1.1 has not been yet resolved experimentally, however, the encoding gene is known (SCN1A gene) (Malo et al., 1994). We used the 3D structure obtained previously using homology modeling (for details see (Montanino et al., 2020)). As template, the cryo-electron microscopy structure of putative sodium channel from American cockroach, NavPaS (PDB ID: 5X0M) (Shen et al., 2017) was used. The structure had 100% confidence (matching probability) and 48% sequence identity (identical residues) with NavPaS. The missing terminal domains and linkers were added and the structure was energy minimized and shortly equilibrated in the PC lipid bilayers at the atomistic level using CHARMM36 force field (Pastor and MacKerell, 2011; Best et al., 2012) before building the CG model.

#### 2.1.3 Force Field

The membrane and protein systems were described at the coarse-grained level using the MARTINI2.2 force field (Marrink et al., 2004; Marrink et al., 2007; Monticelli et al., 2008; de Jong et al., 2013) together with one-bead non-polar water model (Marrink et al., 2007). In the MARTINI model, small groups of atoms (3–4 heavy atoms) are united into beads which interact with each other by means of empirical potentials.

### 2.2 Systems Setup

#### 2.2.1 Myelin Model

The myelin was modelled as a bilayer having the composition reported in Table 1. The initial lipid coordinates were constructed using the 2,000 lipids and an initial box of 24 × 24 × 12 nm. The equilibrated 20 nm bilayer (at 16 μs) was used as template to build a 40 nm bilayer and a multi-bilayers system. The 40 nm bilayer system has a total of 8,000 lipids and was placed in a cubic box of 41 × 41 × 14 nm and solvated by about 138,000 CG water beads. The multi-bilayers system was built using a distance of 1.7 and 1.6 nm between extracellular layers and cytoplasmic layers, respectively, and was placed in a cubic box of 21 × 21 × 48 nm and solvated by about 106,000 CG water beads. To all the systems NaCl was added to mimic the ionic strength at physiological condition (150 mM NaCl).

#### 2.2.2 Node-of-Ranvier Model

A channel concentration of 3,086 channels/μm^2^ was used to describe the node. The bilayer (containing a total of 6,500 lipids) was placed in a cubic box (42 × 42 × 18 nm) and solvated by about 190,000 CG water beads. NaCl was added to mimic the ionic strength at physiological condition (150 mM NaCl). To achieve a concentration of 3,086 channels/μm^2^, 16 copies of proteins were embedded in a larger bilayer (containing 15,000 lipids in a cubic box of 69 × 69 × 20 nm). The proteins were located at 5 nm distance from each other.

### 2.3 Molecular Dynamics Simulations

All MD simulations were performed using the GROMACS simulation package, version 2016 (Abraham et al., 2015) (manual.gromacs.org/2016). The bilayer systems were equilibrated at constant temperature (37°C) and pressure (1 bar). The temperature was held constant using velocity rescale thermostat (Bussi et al., 2009) with a time constant of 1.0 ps. The pressure was held constant using semi-isotropic Parrinello-Rahman barostat (Parrinello and Rahman, 1981) with a time constant of 12 ps (compressibility of 3 × 10^−4^ bar^−1^). The Verlet cutoff scheme (Páll and Hess, 2013) and a timestep of 10 fs (for myelin system) and 15 fs (for protein-membrane system) were used. Periodic boundary conditions were applied. Non-bonded interactions were calculated between all beads using a cutoff of 1.1 nm. Long-range electrostatic interactions were treated using a reaction field potential (Tironi et al., 1995) with switching distance of 1.1 nm in line with MARTINI setting. After a short equilibration, 16 μs were performed for both node-of-Ranvier and 20 nm myelin models, while 25 μs MD simulations were performed for 40 nm and multi-layer myelin model. Supplementary Figure S1 shows equilibration of box dimension and energy within the first 16 μs. Note, the protein positions were kept fixed in only the simulations at equilibrium to allow the relaxation of the lipid distribution.

To evaluate membrane behavior under mechanical stress we performed 6 μs simulations at constant areal strains (NP_*z*_AT ensemble) for the node-of-Ranvier and 40-nm myelin model. No position constrains was applied to the proteins Values < 0.05 were considered for areal strain. The last 4 μs are used for data production. To monitor the pore formation, we first fast pre-deformed the bilayers in *x* direction up to 30% strain (using a deformation speed of 1 × 10^−5^ nm/ps), then starting from 30% strain two independent simulations were performed using slower deformation speeds: 2.5 × 10^−6^ nm/ps and 25 × 10^−6^ nm/ps. Stretching simulations were performed for the node-of-Ranvier and myelin (40 nm and multi-layer) model. No position constrains was applied to the proteins.

### 2.4 Simulation Analysis

To describe the structure of lipid bilayers, we calculated bilayer thickness and number of lipid contacts. Bilayer thickness was calculated using the method proposed by Pandit et al. (2003), Pandit et al. (2004) and implemented in APL@Voro software (Lukat et al., 2013). Using Voronoi tessellation, the methods identifies the normal distance between phosphorus atoms from two leaflets that are “vertical neighbors” of each other.

Lipid contacts were calculated by counting the number of neighboring lipids within 1.5 nm of proteins or lipids by considering the first tail bead after the headgroup of lipid or polar group of cholesterol molecules. Enrichment factors were calculated as (Corradi et al., 2018):

$$Enrichment\left(L\right)=\frac{Ratio{\left(L\right)}_{x}}{Ratio{\left(L\right)}_{bulk}}$$ (1)

In which $\mathrm{Ratio}{\left(L\right)}_{x}=\frac{{\left(\mathrm{no}.L\right)}_{x}}{{\left(\mathrm{tot}\mathrm{.no}\mathrm{.lipids}\right)}_{x}}$ and $\mathrm{Ratio}{\left(L\right)}_{bulk}=\frac{\mathrm{tot}\mathrm{.no}.\left(L\right)}{\mathrm{tot}\mathrm{.no}\mathrm{.lipids}}$ where *x* was chosen 0.7 and 2.1 nm for lipids around the protein in node-of-Ranvier model and 1.5 nm for lipid distribution around each lipid type in myelin model.

The partition coefficient $K_{mem/wat}$ was calculated as ${\left[solute\right]}_{mem}$/${\left[solute\right]}_{wat}$, where [solute] denotes the concentration of water molecules in the lipid bilayers and water solution, respectively. ${\left[water\right]}_{mem}$ was obtained by dividing the average number of water molecules by the lipid bilayers volume, while for the water concentration in water the experimental value (55.5 mol/L) was used. The membrane volume was corrected to account for the presence of the protein.

The software VMD (Humphrey et al., 1996) was used for graphical representations. Reported values were averaged on 5 μs and the errors were obtained by dividing the data production into five parts and calculating the standard error between them, if it was not specified differently.

### 2.5 Mechanical Properties

Area compressibility modulus ($K_{A}$) is defined as the derivative of surface tension as function of the areal strain,

$$K_{A}=\left(\frac{\partial \gamma }{\partial \epsilon_{A}}\right)T$$ (2)

where *γ* is the surface tension and the areal strain ($\epsilon_{A}$) is defined as $\epsilon_{A}=\left(\frac{A}{A_{0}}\right)-1$. Eq. 2 was chosen to be in line with experimental approach (Needham and Nunn, 1990; Rawicz et al., 2000) used on biological membrane. Evans et al. (1976) and Needham and Nunn (1990) observed a linear relation between surface tension for small areal change (<0.05 areal change) in their study on red blood cell and chlosterol-rich membrane, respectively.

To calculate area compressibility modulus, we used NP_*z*_AT simulations at three strain values ($\epsilon_{A}$ < 0.05). We divided production data in 4 parts and calculated the average *γ* and $\epsilon_{A}$ values *γ* is calculated according to the following

$$\gamma =l_{z}\left(P_{zz}-\frac{P_{xx}}{2}-\frac{P_{yy}}{2}\right)$$ (3)

where $P_{xx}$, $P_{yy}$, and $P_{zz}$ are diagonal elements of pressure matrix and $l_{z}$ is the box height along z.

We performed linear regression between the obtained *γ* and $\epsilon_{A}$ values, the slope of the regression line is $K_{A}$ and the standard error of the slope is the standard error of $K_{A}$.

### 2.6 Multiscale Approach

**FIGURE 1:**
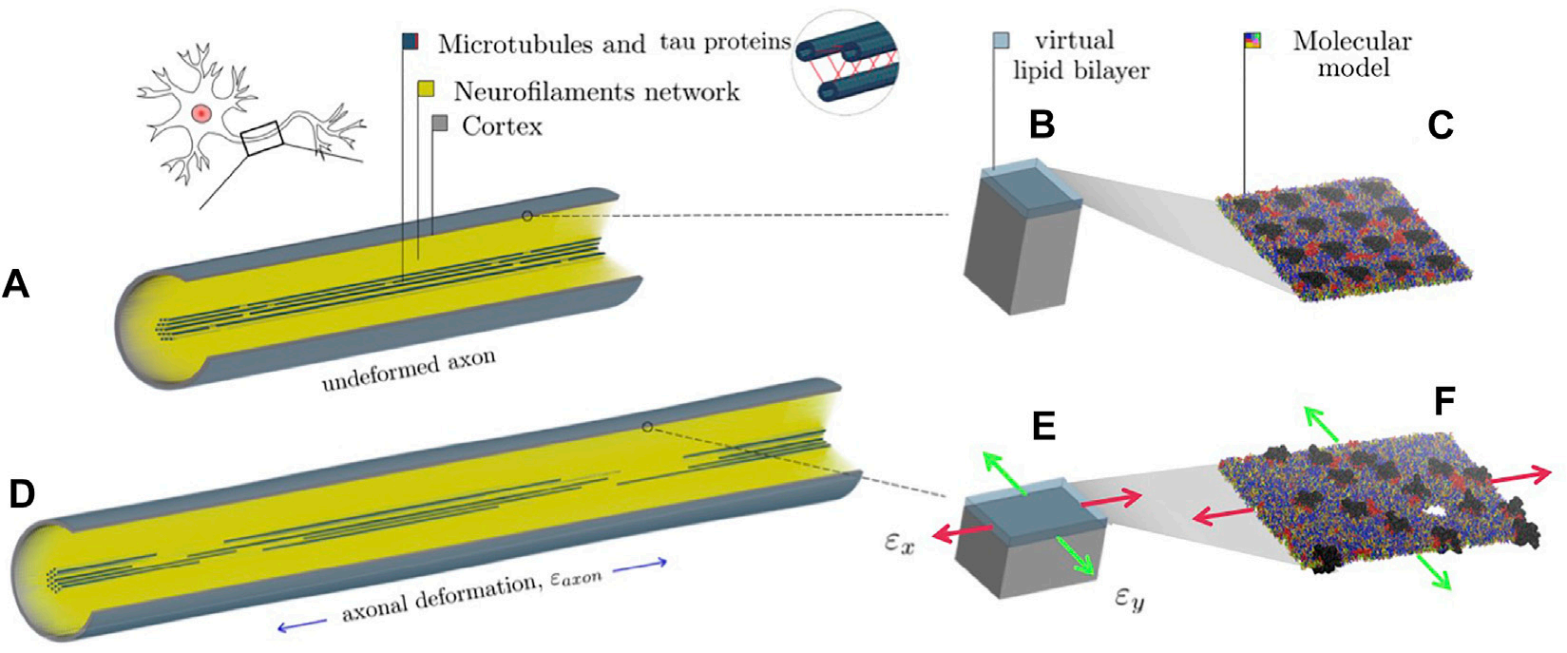
Representation of the combined finite element-molecular based modeling approach (Montanino et al., 2020). **(A)** Finite element axonal model where a quarter of the model was removed to reveal the arrangement of the inner structures. **(B)** Single undeformed cortex element and a juxtaposed virtual lipid bilayer. **(C)** molecular bilayer model. **(D)** The axon finite element model is deformed of a quantity ($\varepsilon_{axon}$). **(E)** Maximum localized deformations ($\varepsilon_{x}\varepsilon_{y}$) are extracted and applied to the molecular model. **(F)** Visualization of the induced pore upon deformation of the molecular model.

To provide insights into the initiation of axonal damage, we combined the FE model of a generic portion of an axon with the molecular-based myelin and node-of-Ranvier models. The node-of-Ranvier model was used to exemplify the approach in Figure 1. Firstly, the axon FE model was utilized to simulate typical stretch injury scenarios, then as a result of axonal deformation, maximum local deformations happening at the cortex level was extracted and applied to the molecular models. The multiscale approach together with details on axon FE simulations was previously described and published in Montanino et al. (2020). The axon FE model (Figure 1C) was validated and described in Montanino and Kleiven (2018). The axon FE model is a 8 μm long representative volume of an axon consisting of three main compartments: a microtubule (MT) bundle, the neurofilament network and the axolemma-cortex complex wrapping the entire structure. To create a general axonal behavior, 10 different FE axon models were generated by randomly moving the MTs discontinuities locations, while keeping the average MTs length of the original model. Details regarding the element and material modelling choices can be found in Montanino and Kleiven (2018). All simulations were performed in LSDYNA using an implicit dynamic solver and the 1st and 2nd principal strains ($\epsilon_{x}$, $\epsilon_{y}$) in the cortex plane were extracted as function of axonal strain for axonal strain rates of 1, 20, and 40/s.

## 3 Results and Discussion

### 3.1 Structural Features of Myelin Model

**FIGURE 2:**
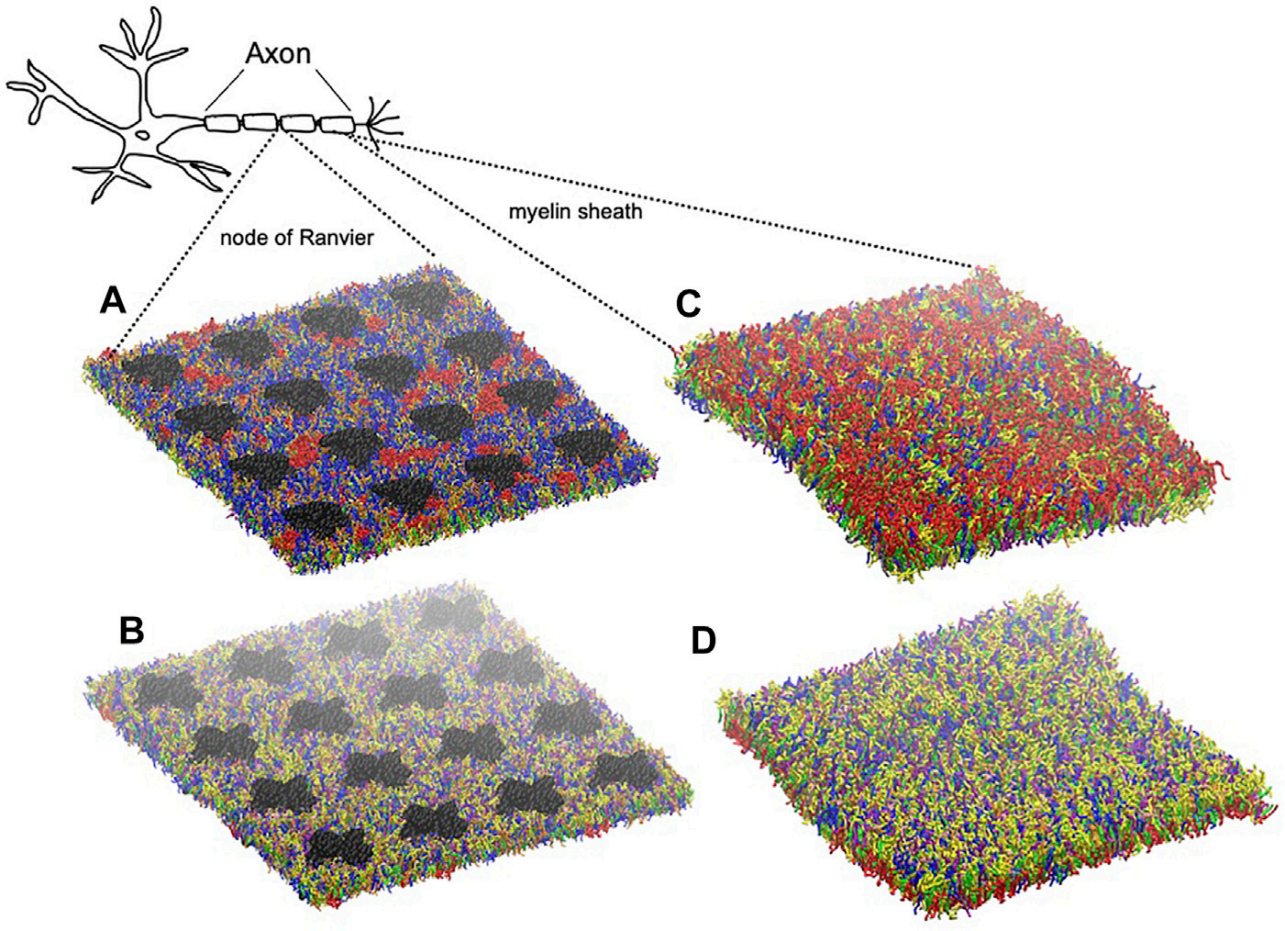
Graphical representation of node-of-Ranvier bilayer model (70 nm) at 16 μs **(A, B)** and myelin bilayer model (40 nm) at 25 μs **(C, D)** together with a draw of a neuron. Both up and down leaflets are shown. Molecules are colored as follows: PC lipids are in blue, PE in yellow, CHOL in green, SM in orange, PS in purple, glycolipids in red, anionic lipids in pink, the rest of lipids in gray, protein in black VDW representation, and water is not visualized for clarity.

The myelin membrane was modelled as a lipid bilayers, having 17 different types of lipids, distributed asymmetrically between two leaflets, labelled as extracellular and cytoplasmic leaflet (Figure 2). The lipids distribution and saturation were assigned accounting for a collection of experimental data on leaflet mole composition, fatty acid length and saturation. In particular, we used the values derived by Inouye and Kirschner (1988b) for myelin in central neurons system to define the lipid disposition in the extracellular and cytoplasmic leaflet; the work of Manzoli et al. (1970) to define the saturation of the lipid tails for phospholipids, while the saturation values for cerebroside were obtained from O’Brien and Rouser (1964) and Bosio et al. (1998). Supplementary Table S1 reports details on the lipid composition of the myelin model together with the experimental reference. In Table 1 we compare the model’s lipids abundance with a wide collection of data on lipid composition observed for myelin sheath in the central nervous system of diverse species (human, bovine, rat). The myelin model has a composition in line with experimental data, small deviations are observed for PE, PS and glycolipid, whose weight fraction is between myelin and axolemma experimental composition.

The myelin bilayers were simulated for 16 μs. Box dimensions and total energy converged in the first microseconds (Supplementary Figure S1). To check the convergence of the lipid distribution, we compare the numbers of lipid neighboring for the 20 and 40 nm bilayers: the values over the last 5 μs agrees within the fluctuations (Supplementary Figure S2).

The equilibrated myelin bilayer has a thickness of 4.51 nm (Table 2), larger than axolemma bilayers and comparable with the value derived using cable theory and the mean dielectric constant of squid axon myelin (around 4.5 nm) (Min et al., 2009). Table 3 reports the lipid enrichment factor. In the extracellular leaflet a depletion of phospholipids (in particular PC and PE, and SM) is observed around glycolipids. A deplection of PI around PI and enrichment of PS around SM are also reported. In the cytoplasmic leaflet no relevant enrichment/depletion is observed, most of the values are with 5% from an homogeneous distribution.

**TABLE 2:** Bilayers structural and mechanical properties. In parenthesis standard error.

Properties Simulated Models	Area compressibility Modulus $K_{A}\left(mN/m\right)$	Thickness Coefficient (*nm*)	Water partition $\text{log}K_{mem/wat}$	Rupture Strain
model for unmyel.axon	352 (3)^a^	4.168 (0.001)^a^	−1.75 (0.01)^a^	0.47^a^; 0.51^a^
node-of-Ranvier model	252 (20)	4.032 (0.001)	−1.50 (0.01)	0.36^b^; 0.40^c^
myelin model	339 (12)	4.506 (0.015)	−1.98 (0.01)	0.70^b^; 0.82^c^

Lowest observed rupture strains are reported for each deformation rate.

a Data from Montanino et al. (2020).

b deformation rate 2.5 × 10^−6^ nm/ps.

c deformation rate 25 × 10^−6^ nm/ps.

**TABLE 3:** Lipd enrichment factor in myelin bilayer. Number of lipids within 1.5 nm of a reference lipid type are reported for the extracellular and cytoplasmic leaflet separately. Standard errors are less than 0.1

Lipids type	PC	PE	PS	SM	PI	Glycolipid	CHOL
Extracellular leaflet							
PC	1.15	1.16	1.15	1.11	1.14	0.84	1.08
PE	1.16	1.14	1.08	1.03	1.07	0.85	1.07
PS	1.12	1.04	0.89	1.17	0.91	0.88	1.07
SM	1.13	1.05	1.23	1.08	1.13	0.88	1.06
PI	1.13	1.06	0.94	1.10	0.38	0.89	1.07
Glycolipid	0.77	0.78	0.83	0.79	0.82	1.03	1.07
Cytosplasmic leaflet							
PC	0.99	1.00	1.03	1.00	1.00	-	1.00
PE	1.00	0.99	1.04	0.97	1.04	-	0.99
PS	1.02	1.02	0.95	1.02	0.94	-	1.00
SM	1.03	0.99	1.06	1.01	1.02	-	0.98
PI	1.01	1.04	0.96	1.01	0.92	-	0.99

### 3.2 Node-of-Ranvier Model

Node of Ranvier was modelled as lipid bilayer with embedded ion channels. We use a plasma membrane lipid bilayers composition. Table 1 compares the composition with lipid abundance observed for axolemma in mammalian neuron system. The values are closer to the composition of axolemma than to the one of myelin. The larger deviation from experimental values is observed for the glycolipids. We have also to note that we can not exclude that the experimental composition for axolemma may be contaminated by myelin lipids (DeVries et al., 1981). In comparison with the composition used to describe myelin sheath, model for node-of-Ranvier is characterized by a lower content of glycolipids and CHOL molecules and higher content of PC and SM lipids, in line with the trend shown by experimental values. The model is also characterized by having shorter long-chain fatty acids than myelin model in line with experimental observation (DeVries et al., 1981).

To describe the proteins in the node, we use sodium channel protein type subunit alpha (Nav1.1), a characteristic protein of nodal portions of myelinates axons, belonging to the family of voltage-gated ion channels (Duflocq et al., 2008). It is known that several different ion channels are present, but currently information are available to model solely sodium channel protein type subunit alpha (Nav1.1). 16 proteins were embedded in the bilayers (equivalent to 3,000 channels/μm^2^) to mimic a value of ion channel concentration in line with the experimental observation for the node of Ranvier (Wann, 1993; Hu and Jonas, 2014). Below we compare the results with previously performed simulations of a bilayer, having the same lipid composition as the node but only one embedded protein, representing a condition closer to axolemma in unmyelinated axon (Montanino et al., 2020).

We checked the convergence of lipid distribution around the proteins by monitoring the lipid-protein contacts (Supplementary Figure S3). In general, the lipid-protein contacts are in overall agreement with the contact recorded for one protein embedded in the bilayer. The node-of-Ranvier bilayer has a thickness of around 4.0 nm (Table 2). High protein density makes the bilayer slightly thinner: a values of 4.2 was calculated for the bilayer with one embedded protein. The difference in lipid composition (both in tail length and head-group) between the node-of-Ranvier and myelin bilayers results difference in the thickness and the water permeability, given the proportionality between the partition coefficient $K_{mem/wat}$ and the permeability ($P_{m}$). The myelin is thicker and is less permeable to water than the node of Ranvier. Now we can proceed to evaluate what is the effect of the composition on the mechanical feature.

### 3.3 Bilayer Models Under Deformation

**FIGURE 3:**
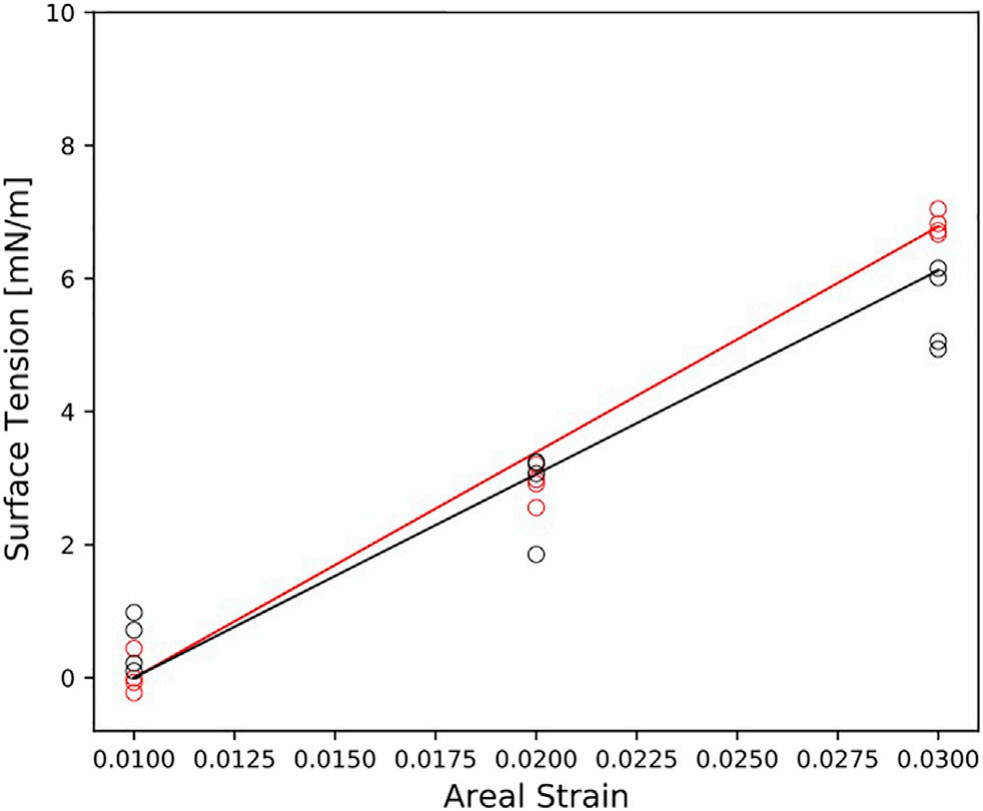
Surface tension as function of the areal strain for myelin and node-of-Ranvier model, colored in red and black respectively. Reported data (circles) corresponds to the average on 1 μs time-window. The slope of the line is $K_{A}$.

To study the bilayers response to mechanical deformation, we performed simulations at three different areal strains. Figure 3 reports the surface tension as a function of the areal strain for each membrane model. In general, the surface tension increases linearly for small areal strain. The myelin model shows the steepest slope of surface tension-areal strain (Figure 3). That means that the myelin model is more resistant to change its area than node-of-Ranvier model.

The area compressibility modulus, $K_{A}$, was estimated for small areal strain values (less than 0.05) and reported in Table 2. A larger $K_{A}$ values is observed for the myelin model than for node-of-Ranvier models: a value of 339 mN/m and 252 mN/m, respectively for myelin and node-of-Ranvier bilayers, was obtained. At this point we can not distinguish if the difference is due to the protein concentration, lipid composition or a combination of them. The value for the axolemma model in unmyelinated (plasma membrane with one embedded protein) is in line with the area compressibility modulus measured for red blood cell membranes (375 ± 60 mN/m) at 37°C by Waugh and Evans (1979). Unlucky a validation for all the models toward experiments is not possible due to lack of experimental data. A previous study (Saeedimasine et al., 2019) shows that atomistic and CG simulations give different $K_{A}$ values, but similar trend upon change in lipid composition. That gives us trust in the observed trend, even if the absolute values can not be validated.

**FIGURE 4:**
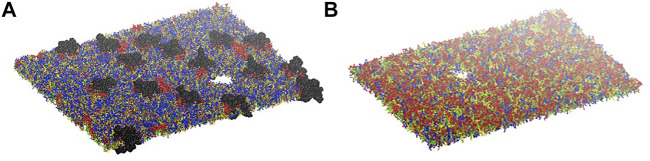
Node-of-Ranvier **(A)** and myelin **(B)** bilayers at 0.36 and 0.70 strain, respectively. For color code see Figure 2.

Simulations at different deformation rate were performed to identify the rupture points. We define the rupture point when at least one pore is formed in the bilayer. To detect the formation we monitored the surface tension: a jump in the surface tension corresponds to the formation of at least one pore. In the myelin model pores were observed at 0.7–0.8 strain while in the node-of-Ranvier model at around 0.4 (Figure 4). Poration in both bilayers occurs in region lacking protein and glycolipids. This type of lipids are mainly found in the outer leaflet of membranes and are known to make the bilayer more resistant to deformation thanks to interactions between sugar headgroups (Saeedimasine et al., 2019).

**FIGURE 5:**
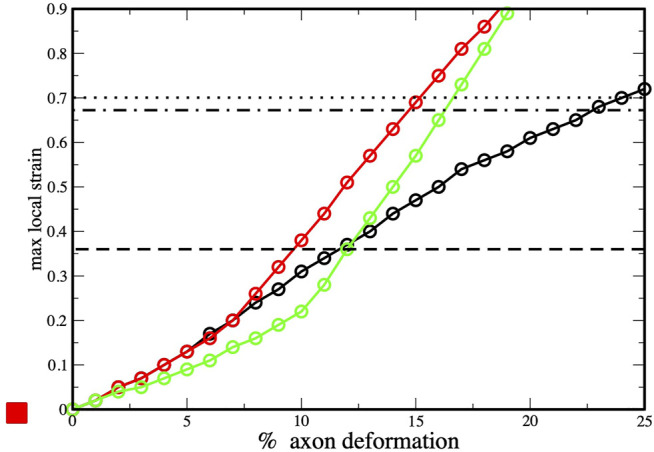
Axonal deformation versus maximum local strains at three strain rates: 1 s^−1^ (black), 10 s^−1^ (red) and 40 s^−1^ (green). Values obtained from finite element simulations of axonal stretch (Montanino et al., 2020). The dotted line corresponds to rupture strain observed for myelin model (and dashed-dot lines to the myelin multi-layer model), while the dashed line corresponds the to rupture strain observed for the node-of-Ranvier model.

To provide insights into the initiation of axonal damage, we combined the FE model of a generic portion of an axon with the molecular-based myelin and node-of-Ranvier models. Firstly, the axon FE model was utilized to simulate typical stretch injury scenarios, then as a result of axonal deformation, maximum local deformations happening at the cortex level was extracted and applied to the molecular models (Figure 1). Figure 5 shows the relation between the applied axon deformation and the resulting maximum local deformation observed at different strain rates. We found that higher strain rates do not lead to higher local maximum strains for axonal strains less than 12%. This is due to the viscoelastic properties of tau proteins and of the cortex itself resulting in less cortex deformation at strain rate 40/s compared to 1 or 20/s (Montanino et al., 2020).

We connect the bilayer strain at which local events occurs in molecular model (e.i pore formation) with the maximum local strain observed in the finite element simulations during axonal deformation. This allows to understand which axonal strain correspond to the bilayer rupture. The node-of-Ranvier model can withstand an applied deformation up to 36%, corresponding to axonal deformation of 9–11% (see dashed line in Figure 5). At higher strains pore formation is observed. While poration occurs at a larger cortex strain (70%) for the myelin model and this corresponds to an axonal deformation of 15–23%.

**FIGURE 6:**
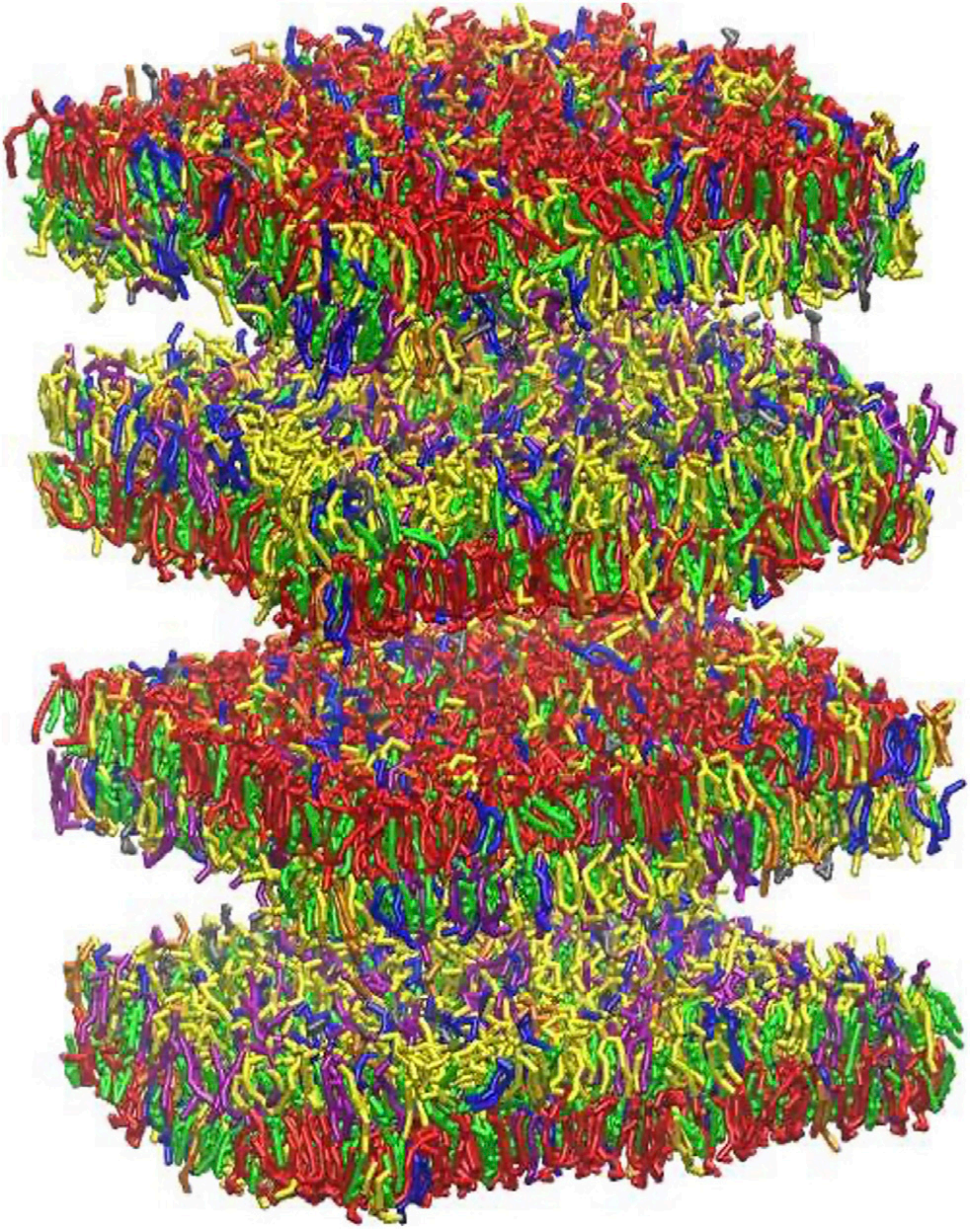
Graphical representation of multi-layer myelin at 25 μs. The multi-bilayers system was built using a distance of around 1.7 nm between extracellular and cytoplasmic layers. For lipid molecules color code see Figure 2 and for bilayer composition see Table 2.

Myelin sheath consists of not one bilayer but of repeating units of double bilayers separated by aqueous layers. To verify the effect of the multi layers structure on the rupture threshold, we have built a double bilayers myelin model (Figure 6), equilibrated and deformed it as it was done for the single bilayer model. The distances between the bilayers were taken from X-ray diffraction data (Caspar and Kirschner, 1971; Kirschner et al., 1989): 1.6 and 1.7 nm were reported as widths of the spaces between membranes at the cytoplasmic and extracellular appositions, respectively, for the central nervous system. Starting from a strain value of 0.66 pore formation can occur in one of the bilayers (Figure 6). The first pore formation is followed by formation of multiple pores in different layers (at strain 0.73). This corresponds to an axonal strain of 14–22%, in line of what observed for the single-bilayer myelin model.

All in all, our results show that mechanoporation occurs at 49–74% higher strain for the myelin and at 15–23% lower strain at the node of Ranvier compared with axolemma with one embedded protein. *In vivo* and *ex vivo* experiments on uniaxially oriented neuronal cell and spinal nerve showed injurious changes above 10% applied strain (Singh et al., 2015; Nakadate et al., 2017). Experiments conducted on non-oriented neuronal culture indicated that mechanoporation occurs only when strains higher than 10% were uniaxially applied to the culture (Hemphill et al., 2011). If mechanico-poration trigger axonal brain injury, our results indicate that such events in myelined axon will occur at the level of the node of Ranvier. Indeed, according to our models, poration in the node-of-Ranvier occurs at axonal strains going from 9% to 11%, while myelin rupture occurs at axonal strains larger than 15%.

Although our modeling approach brings some insights into the axonal injury mechanism, note that we do not aim to describe the injury event, since those events occur at time scale (fraction of seconds) not accessible to current molecular simulations. Moreover, the simulated bilayers are simplified models of cellular membrane: they do not account for all the different embedded proteins, they are described by a coarse grained model (where a group of atoms is described by a particle). Finally, the model does not account for possible protein structural change at different strains, since the protein is described as a semi-rigid body.

## 4 Conclusion

In this study, we combine CG molecular dynamics simulations and FE method to improve our understanding of what can trigger axonal brain injury. To achieve this we used molecular models to describe sub-cellular components of the myelinated axon, in particular myelin sheath and node of Ranvier, and a FE simulations of the axon.

The ex-novo built myelin model well reproduces the myelin experimental composition and structural feature of myelin. The model is more glycolipid-rich with longer fatty acid tails than the model used to describe the node-of-Ranvier. The myelin bilayer has a higher thickness, rupture point and area compressibility modulus than node of Ranvier. The results support a scenario where node-of-ranvier is most vulnerable component, myelin has more of a supporting role given that it likely will not porate before the axolemma, in unmyelined axon, and node-of-Ranvier do.

Combining the results with finite element simulations of the axon model, we provided quantitative evidences that mechanoporation of the axon membranes is an event that cannot be excluded in a typical axonal injury scenario. Our results indicate that mechanoporation may occur at the node of Ranvier at the thresholds proposed in the literature for axonal injury, while no myelin rupture is observed at the injury threshold. Poration occurs at more than 50% higher strain for the myelin compared with node-of-Ranvier and axolemma (in unmyelinated axon). The findings contribute to increase our knowledge of axonal sub-cellular components and help to understand better the mechanism behind axonal brain injury. Finally, our work shows the power of combining FE and MD to describe complex biological scenario, that requires the description of different length scale.

## Data Availability

The original contributions presented in the study are included in the article/Supplementary Material, further inquiries can be directed to the corresponding author. Topology and coordinate files for myelin model are deposited in https://github.com/alevil-gmx/myelin_model.

## References

[B1] Abraham M. J. Murtola T. Schulz R. Páll S. Smith J. C. Hess B. (2015). GROMACS: High Performance Molecular Simulations through Multi-Level Parallelism from Laptops to Supercomputers. SoftwareX 1–2, 19–25. 10.1016/j.softx.2015.06.001

[B2] Bain A. C. Meaney D. F. (2000). Tissue-Level Thresholds for Axonal Damage in an Experimental Model of Central Nervous System White Matter Injury. J. Biomechanical Eng. 122, 615–622. 10.1115/1.1324667 11192383

[B3] Best R. B. Zhu X. Shim J. Lopes P. E. M. Mittal J. Feig M. (2012). Optimization of the Additive CHARMM All-Atom Protein Force Field Targeting Improved Sampling of the Backbone ϕ, ψ and Side-Chain χ1 and χ2 Dihedral Angles. J. Chem. Theor. Comput. 8, 3257–3273. 10.1021/ct300400x PMC3549273 23341755

[B4] Bosio A. Binczek E. Haupt W. F. Stoffel W. (1998). Composition and Biophysical Properties of Myelin Lipid Define the Neurological Defects in Galactocerebroside- and Sulfatide-Deficient Mice. J. Neurochem. 70, 308–315. 10.1046/j.1471-4159.1998.70010308.x 9422376

[B5] Bussi G. Zykova-Timan T. Parrinello M. (2009). Isothermal-isobaric Molecular Dynamics Using Stochastic Velocity Rescaling. J. Chem. Phys. 130, 074101. 10.1063/1.3073889 19239278

[B6] Camejo G. Villegas G. M. Barnola F. V. Villegas R. (1969). Characterization of Two Different Membrane Fractions Isolated from the First Stellar Nerves of the Squid Dosidicus Gigas. Biochim. Biophys. Acta (Bba) - Biomembranes 193, 247–259. 10.1016/0005-2736(69)90186-2 4242763

[B7] Caspar D. L. D. Kirschner D. A. (1971). Myelin Membrane Structure at 10 Å Resolution. Nat. New Biol. 231, 46–52. 10.1038/newbio231046a0 5283387

[B8] Catterall W. A. Goldin A. L. Waxman S. G. (2005). International Union of Pharmacology. XLVII. Nomenclature and Structure-Function Relationships of Voltage-Gated Sodium Channels. Pharmacol. Rev. 57, 397–409. 10.1124/pr.57.4.4 16382098

[B9] Corradi V. Mendez-Villuendas E. Ingólfsson H. I. Gu R.-X. Siuda I. Melo M. N. (2018). Lipid-Protein Interactions Are Unique Fingerprints for Membrane Proteins. ACS Cent. Sci. 4, 709–717. 10.1021/acscentsci.8b00143 29974066 PMC6028153

[B10] de Jong D. H. Singh G. Bennett W. F. D. Arnarez C. Wassenaar T. A. Schäfer L. V. (2013). Improved Parameters for the Martini Coarse-Grained Protein Force Field. J. Chem. Theor. Comput. 9, 687–697. 10.1021/ct300646g 26589065

[B11] DeVries G. H. Campbell B. Saunders R. (1999). Isolation and Characterization of Unmyelinated Axolemma from Bovine Splenic Nerve. J. Neurosci. Res. 57, 670–679. 10.1002/(sici)1097-4547(19990901)57:5<670::aid-jnr9>3.0.co;2-b 10462691

[B12] DeVries G. H. Zetusky W. J. Zmachinski C. Calabrese V. P. (1981). Lipid Composition of Axolemma-Enriched Fractions from Human Brains. J. Lipid Res. 22, 208–216. 10.1016/s0022-2275(20)35364-5 7240954

[B13] Duflocq A. Le Bras B. Bullier E. Couraud F. Davenne M. (2008). Nav1.1 Is Predominantly Expressed in Nodes of Ranvier and Axon Initial Segments. Mol. Cell Neurosci. 39, 180–192. 10.1016/j.mcn.2008.06.008 18621130

[B14] Evans E. A. Waugh R. Melnik L. (1976). Elastic Area Compressibility Modulus of Red Cell Membrane. Biophysical J. 16, 585–595. 10.1016/S0006-3495(76)85713-X PMC1334882 1276386

[B15] Fitzpatrick M. O. Maxwell W. L. Graham D. I. (1998). The Role of the Axolemma in the Initiation of Traumatically Induced Axonal Injury. J. Neurol. Neurosurg. Psychiatry 64, 285–287. 10.1136/jnnp.64.3.285 9527135 PMC2169978

[B16] Hemphill M. A. Dabiri B. E. Gabriele S. Kerscher L. Franck C. Goss J. A. (2011). A Possible Role for Integrin Signaling in Diffuse Axonal Injury. PLOS ONE 6, e22899–11. 10.1371/journal.pone.0022899 21799943 PMC3142195

[B17] Hill C. S. Coleman M. P. Menon D. K. (2016). Traumatic Axonal Injury: Mechanisms and Translational Opportunities. Trends Neurosciences 39, 311–324. 10.1016/j.tins.2016.03.002 PMC5405046 27040729

[B18] Hu H. Jonas P. (2014). A Supercritical Density of Na+ Channels Ensures Fast Signaling in GABAergic Interneuron Axons. Nat. Neurosci. 17, 686–693. 10.1038/nn.3678 24657965 PMC4286295

[B19] Humphrey W. Dalke A. Schulten K. (1996). VMD: Visual Molecular Dynamics. J. Mol. Graphics 14, 33–38. 10.1016/0263-7855(96)00018-5 8744570

[B20] Ingólfsson H. I. Melo M. N. Van Eerden F. J. Arnarez C. Lopez C. A. Wassenaar T. A. (2014). Lipid Organization of the Plasma Membrane. J. Am. Chem. Soc. 136, 14554–14559. 10.1021/ja507832e 25229711

[B21] Inouye H. Kirschner D. A. (1988b). Membrane Interactions in Nerve Myelin: II. Determination of Surface Charge from Biochemical Data. Biophysical J. 53, 247–260. 10.1016/S0006-3495(88)83086-8 PMC1330145 3345333

[B22] Inouye H. Kirschner D. A. (1988a). Membrane Interactions in Nerve Myelin. I. Determination of Surface Charge from Effects of pH and Ionic Strength on Period. Biophysical J. 53, 235–245. 10.1016/S0006-3495(88)83085-6 PMC1330144 3345332

[B23] Iwata A. (2004). Traumatic Axonal Injury Induces Proteolytic Cleavage of the Voltage-Gated Sodium Channels Modulated by Tetrodotoxin and Protease Inhibitors. J. Neurosci. 24, 4605–4613. 10.1523/jneurosci.0515-03.2004 15140932 PMC6729402

[B24] Johnson V. E. Stewart W. Smith D. H. (2013). Axonal Pathology in Traumatic Brain Injury. Exp. Neurol. 246, 35–43. 10.1016/j.expneurol.2012.01.013 22285252 PMC3979341

[B25] Kirschner D. A. Inouye H. Ganser A. L. Mann V. (1989). Myelin Membrane Structure and Composition Correlated: A Phylogenetic Study. J. Neurochem. 53, 1599–1609. 10.1111/j.1471-4159.1989.tb08558.x 2795020

[B26] LaPlaca M. C. Thibault L. E. (1997). Anin Vitro Traumatic Injury Model to Examine the Response of Neurons to a Hydrodynamically-Induced Deformation. Ann. Biomed. Eng. 25, 665–677. 10.1007/BF02684844 9236979

[B27] Lukat G. Krüger J. Sommer B. (2013). APL@voro: A Voronoi-Based Membrane Analysis Tool for GROMACS Trajectories. J. Chem. Inf. Model. 53, 2908–2925. 10.1021/ci400172g 24175728

[B28] Malo M. S. Blanchard B. J. Andresen J. M. Srivastava K. Chen X.-N. Li X. (1994). Localization of a Putative Human Brain Sodium Channel Gene (SCN1A) to Chromosome Band 2q24. Cytogenet. Cel Genet 67, 178–186. 10.1159/000133818 8062593

[B29] Manzoli F. A. Stefoni S. Manzoli-Guidotti L. Barbieri M. (1970). The Fatty Acids of Myelin Phospholipids. FEBS Lett. 10, 317–320. 10.1016/0014-5793(70)80462-8 11945422

[B30] Marrink S. J. de Vries A. H. Mark A. E. (2004). Coarse Grained Model for Semiquantitative Lipid Simulations. J. Phys. Chem. B 108, 750–760. 10.1021/jp036508g

[B31] Marrink S. J. Risselada H. J. Yefimov S. Tieleman D. P. De Vries A. H. (2007). The MARTINI Force Field: Coarse Grained Model for Biomolecular Simulations. J. Phys. Chem. B 111, 7812–7824. 10.1021/jp071097f 17569554

[B32] Maxwell W. (2013). Damage to Myelin and Oligodendrocytes: a Role in Chronic Outcomes Following Traumatic Brain Injury? Brain Sci. 3, 1374–1394. 10.3390/brainsci3031374 24961533 PMC4061868

[B33] Menon D. K. Schwab K. Wright D. W. Maas A. I. (2010). Position Statement: Definition of Traumatic Brain Injury. Arch. Phys. Med. Rehabil. 91, 1637–1640. 10.1016/j.apmr.2010.05.017 21044706

[B34] Mierzwa A. J. Marion C. M. Sullivan G. M. McDaniel D. P. Armstrong R. C. (2015). Components of Myelin Damage and Repair in the Progression of White Matter Pathology after Mild Traumatic Brain Injury. J. Neuropathol. Exp. Neurol. 74, 218–232. 10.1097/nen.0000000000000165 25668562 PMC4327393

[B35] Min Y. Kristiansen K. Boggs J. M. Husted C. Zasadzinski J. a. Israelachvili J. (2009). Interaction Forces and Adhesion of Supported Myelin Lipid Bilayers Modulated by Myelin Basic Protein. Proc. Natl. Acad. Sci. 106, 3154–3159. 10.1073/pnas.0813110106 19218452 PMC2651331

[B36] Montanino A. Kleiven S. (2018). Utilizing a Structural Mechanics Approach to Assess the Primary Effects of Injury Loads onto the Axon and its Components. Front. Neurol. 9, 643. 10.3389/fneur.2018.00643 30127763 PMC6087765

[B37] Montanino A. Saeedimasine M. Villa A. Kleiven S. (2020). Localized Axolemma Deformations Suggest Mechanoporation as Axonal Injury Trigger. Front. Neurol. 11, 25. 10.3389/fneur.2020.00025 32082244 PMC7005088

[B38] Monticelli L. Kandasamy S. K. Periole X. Larson R. G. Tieleman D. P. Marrink S.-J. (2008). The MARTINI Coarse-Grained Force Field: Extension to Proteins. J. Chem. Theor. Comput. 4, 819–834. 10.1021/ct700324x 26621095

[B39] Mu J. Li M. Wang T. Li X. Bai M. Zhang G. (2019). Myelin Damage in Diffuse Axonal Injury. Front. Neurosci. 13, 217. 10.3389/fnins.2019.00217 30941005 PMC6433984

[B40] Nakadate H. Kurtoglu E. Furukawa H. Oikawa S. Aomura S. Kakuta A. (2017). “Strain-rate Dependency of Axonal Tolerance for Uniaxial Stretching,” in 61st Stapp Car Crash Conference, November 8–10, 2021, Denver, CO (The Stapp Association). 10.4271/2017-22-0003 29394435

[B41] Needham D. Nunn R. S. (1990). Elastic Deformation and Failure of Lipid Bilayer Membranes Containing Cholesterol. Biophysical J. 58, 997–1009. 10.1016/S0006-3495(90)82444-9 PMC1281045 2249000

[B42] O’Brien J. S. Rouser G. (1964). The Fatty Acid Composition of Brain Sphingolipids: Sphingomyelin, Ceramide, Cerebroside, and Cerebroside Sulfate. J. Lipid Res. 5, 339–342. 5873370

[B43] Páll S. Hess B. (2013). A Flexible Algorithm for Calculating Pair Interactions on SIMD Architectures. Computer Phys. Commun. 184, 2641–2650. 10.1016/j.cpc.2013.06.003

[B44] Pandit S. A. Bostick D. Berkowitz M. L. (2003). An Algorithm to Describe Molecular Scale Rugged Surfaces and its Application to the Study of a Water/lipid Bilayer Interface. J. Chem. Phys. 119, 2199–2205. 10.1063/1.1582833

[B45] Pandit S. A. Vasudevan S. Chiu S. W. Jay Mashl R. Jakobsson E. Scott H. L. (2004). Sphingomyelin-cholesterol Domains in Phospholipid Membranes: Atomistic Simulation. Biophysical J. 87, 1092–1100. 10.1529/biophysj.104.041939 PMC1304449 15298913

[B46] Parrinello M. Rahman A. (1981). Polymorphic Transitions in Single Crystals: A New Molecular Dynamics Method. J. Appl. Phys. 52, 7182–7190. 10.1063/1.328693

[B47] Pastor R. W. MacKerell A. D. (2011). Development of the CHARMM Force Field for Lipids. J. Phys. Chem. Lett. 2, 1526–1532. 10.1021/jz200167q 21760975 PMC3133452

[B48] Pettus E. H. Povlishock J. T. (1996). Characterization of a Distinct Set of Intra-axonal Ultrastructural Changes Associated with Traumatically Induced Alteration in Axolemmal Permeability. Brain Res. 722, 1–11. 10.1016/0006-8993(96)00113-8 8813344

[B49] Rasband M. N. Macklin W. B. (2012). “Myelin Structure and Biochemistry,” in Basic Neurochemistry. Editors Brady S. T. Siegel G. J. Albers R. W. Price D. L. B. T. Eighth Edition (New York: Academic Press), 180, 199. 10.1016/B978-0-12-374947-5.00010-9

[B50] Rawicz W. Olbrich K. C. McIntosh T. Needham D. Evans E. (2000). Effect of Chain Length and Unsaturation on Elasticity of Lipid Bilayers. Biophysical J. 79, 328–339. 10.1016/S0006-3495(00)76295-3 PMC1300937 10866959

[B51] Saeedimasine M. Montanino A. Kleiven S. Villa A. (2019). Role of Lipid Composition on the Structural and Mechanical Features of Axonal Membranes: a Molecular Simulation Study. Sci. Rep. 9, 8000. 10.1038/s41598-019-44318-9 31142762 PMC6541598

[B52] Shen H. Zhou Q. Pan X. Li Z. Wu J. Yan N. (2017). Structure of a Eukaryotic Voltage-Gated Sodium Channel at Near-Atomic Resolution. Science 355, eaal4326. 10.1126/science.aal4326 28183995

[B53] Singh S. Pelegri A. A. Shreiber D. I. (2015). Characterization of the Three-Dimensional Kinematic Behavior of Axons in Central Nervous System White Matter. Biomech. Model. Mechanobiol 14, 1303–1315. 10.1007/s10237-015-0675-z 25910712

[B54] Tang-Schomer M. D. Johnson V. E. Baas P. W. Stewart W. Smith D. H. (2012). Partial Interruption of Axonal Transport Due to Microtubule Breakage Accounts for the Formation of Periodic Varicosities after Traumatic Axonal Injury. Exp. Neurol. 233, 364–372. 10.1016/j.expneurol.2011.10.030 22079153 PMC3979336

[B55] Tironi I. G. Sperb R. Smith P. E. van Gunsteren W. F. (1995). A Generalized Reaction Field Method for Molecular Dynamics Simulations. J. Chem. Phys. 102, 5451–5459. 10.1063/1.469273

[B56] Wann K. T. (1993). Neuronal Sodium and Potassium Channels: Structure and Function. Br. J. Anaesth. 71, 2–14. 10.1093/bja/71.1.2 8393689

[B57] Waugh R. Evans E. A. (1979). Thermoelasticity of Red Blood Cell Membrane. Biophysical J. 26, 115–131. 10.1016/S0006-3495(79)85239-X PMC1328507 262408

[B58] Westenbroek R. E. Merrick D. K. Catterall W. A. (1989). Differential Subcellular Localization of the Ri and Rii Na+ Channel Subtypes in Central Neurons. Neuron 3, 695–704. 10.1016/0896-6273(89)90238-9 2561976

[B59] Williams K. A. Deber C. M. Klrschner O. A. (1993). The Structure and Function of Central Nervous System Myelin. Crit. Rev. Clin. Lab. Sci. 30, 29–64. 10.3109/10408369309084665 7683887

[B60] Zambrano F. Cellino M. Canessa-Fischer M. (1971). The Molecular Organization of Nerve Membranes. J. Membrain Biol. 6, 289–303. 10.1007/BF02116575 24177445

