# Supplementary material for: Elucidating Axonal Injuries Through Molecular Modelling of Myelin Sheaths and Nodes of Ranvier
Source: Front Mol Biosci. 2021 Jun 23;8:669897. doi: 10.3389/fmolb.2021.669897 (PMC8260694; doi:10.3389/fmolb.2021.669897)
Supplement: Supplementary file 1 [file DataSheet1.PDF]

Elucidating axonal injuries through molecular modelling of myelin sheaths and nodes of Ranvier

Marzieh Saeedimasine,[1,+] Annaclaudia Montanino,[2] SveinKleiven,[2] Alessandra Villa[3,*]
[1] Department of Biosciences and Nutrition, Karolinska Institutet, Huddinge, Sweden
[2] Division of Neuronic Engineering KTH-Royal Institute of Technology, Stockholm,Sweden
[3] PCD-Center for high performance computing - KTH-Royal Institute of Technology,Stockholm, Sweden
* Correspondence: Alessandra Villa, contact address: avilla@kth.se
+ Present address: Department of Materials and Environmental Chemistry, Stockholm University, Stockholm, Sweden

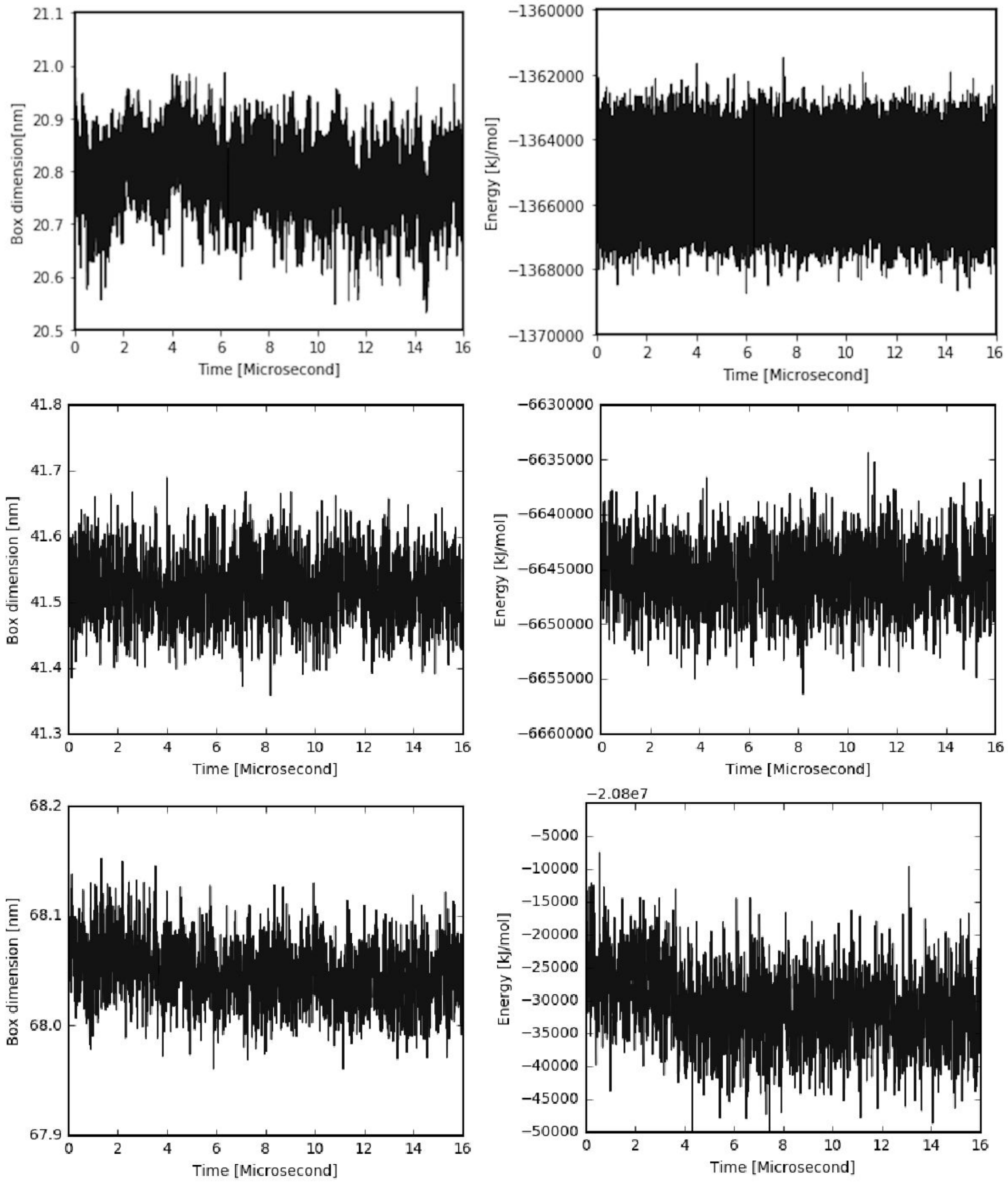

Figure S1: X-dimension and energy time series for (top) myelin model (20-nm), axolemma model with one embedded protein (middle) axolemma with 16 embedded proteins (bottom).

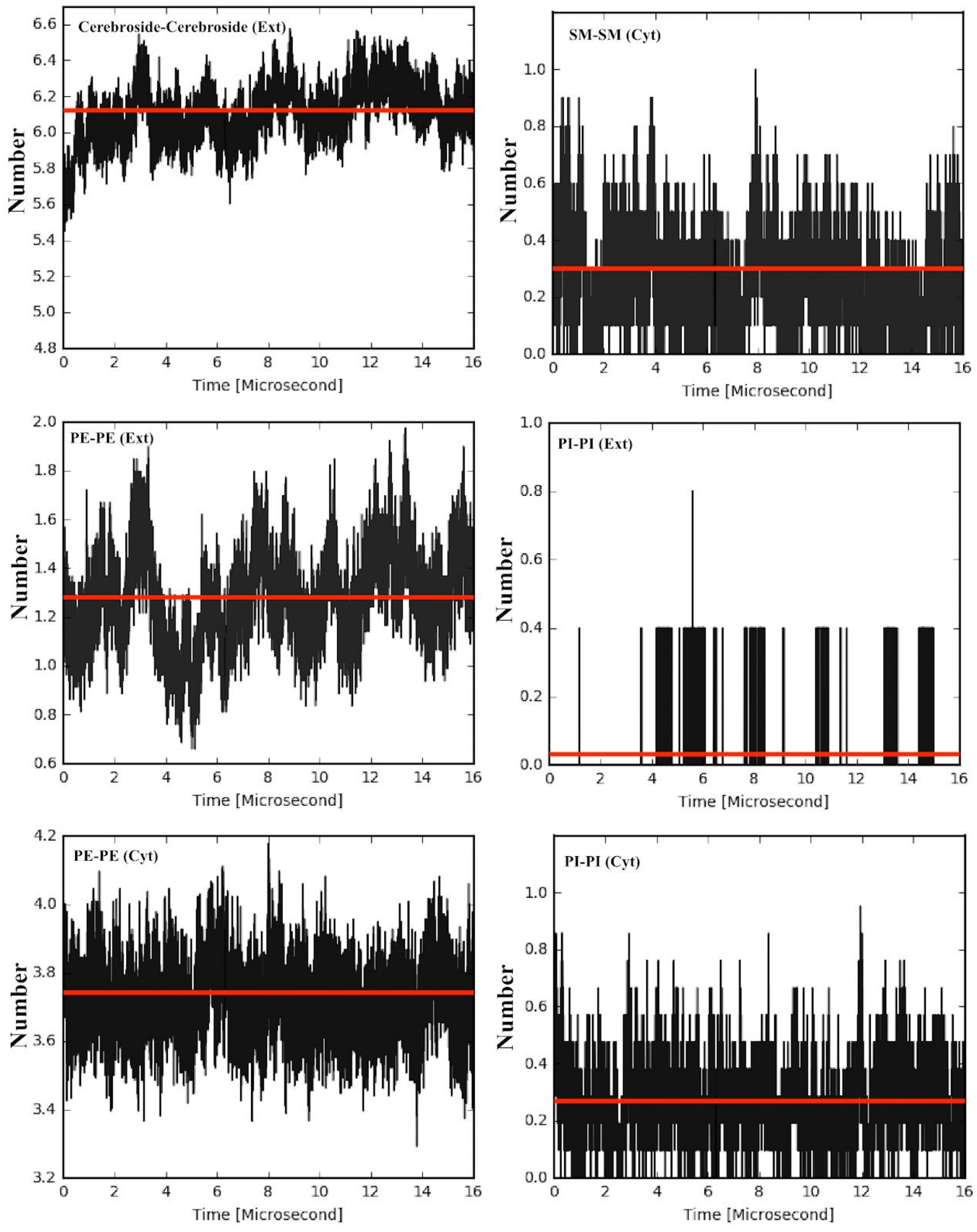

Figure S2: Time series of number of lipid neighboring within 1.5 nm for the 20-nm myelin bilayer model. For comparison the average value on the last 5 μs for 40-nm myelin bilayer model is reported in red.

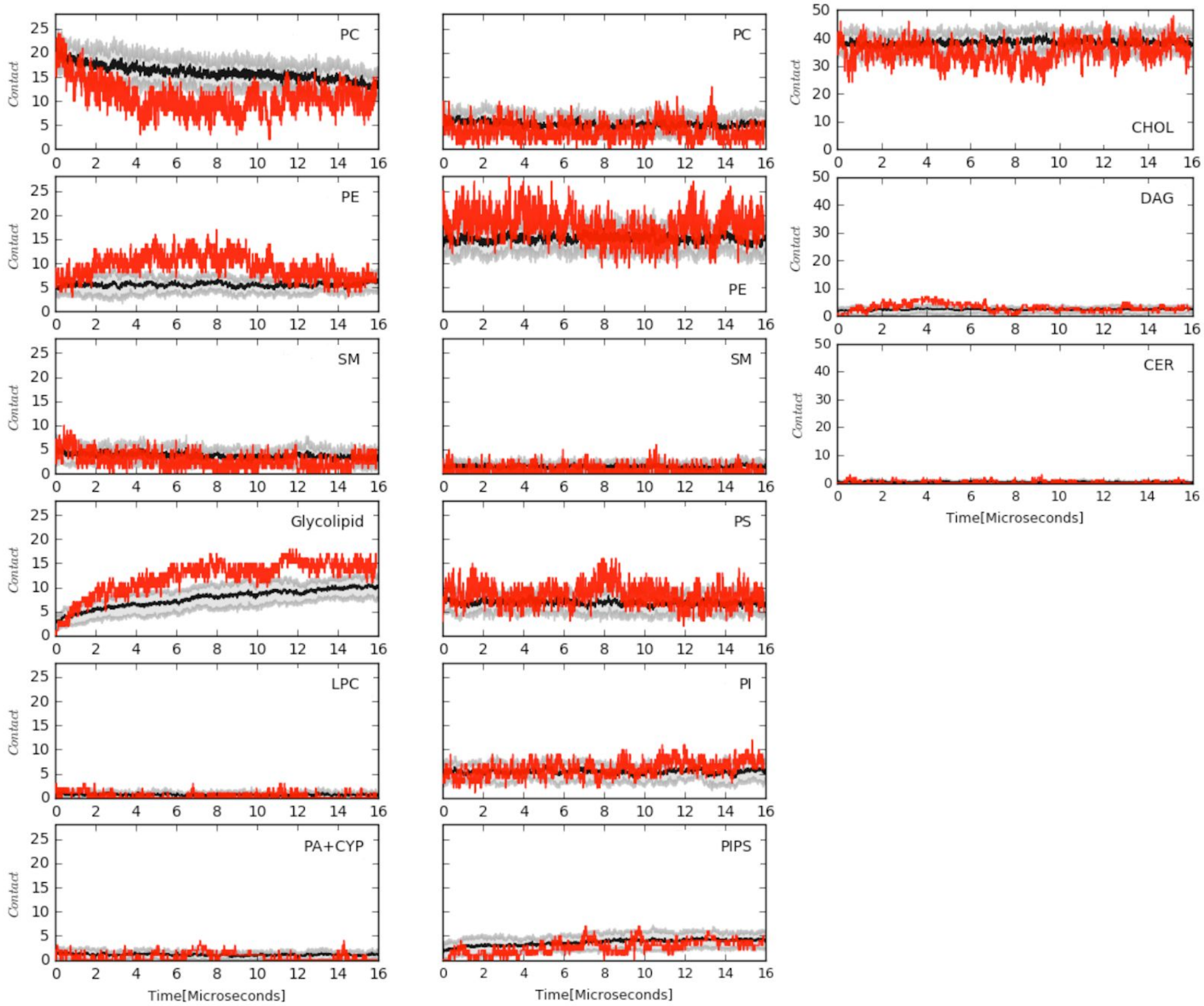

Figure S3: Time series of lipid-protein contacts within 0.7 nm from the Nav1.1 protein. Value for axolemma with one protein embedded in red (Montanino, 2020) and with 16 protein embedded in black (averaged over 16 protein) and standard deviation in grey.

Table S1 Lipids composition, distribution and saturation for myelin bilayer model. Composition values are mol % , saturation values are reported as a fraction between unsaturated and saturated lipid mol.

Lipid type	Extracellular face		Cytoplasmic face		saturated/un saturated	
	model	exp (a)	model	exp(a)	model	exp
**Phosphatidylcholine (PC)**	**7.4**	**7.4**	**11.5**	**11.5**	**1.85**	**1.85**(b)
DPPC 16:0-18:0	2.8		4.4			
POPC 16:0-18:1	3.7		5.7			
DOPC 16:1-18:1	0.9		1.4			
**Phosphatidylethanolamine (PE)**	**7.9**	**7.9**	**27.1**	**27.1**	**0.67**	**0.61**(b)
POPE 16:1-18:1	7.3		24.9			
DUPE 20:5-22:6	0.6		2.2			
**Phosphatidylserine (PS)**	**1.5**	**1.5**	**12.5**	**12.5**	**0.72**	**0.73**(b)
DPPS 16:0-18:0	0.6		5.3			
POPS 16:1-18:1	0.9		7.2			
**Sphingomyelin (SM)**	**2.0**	**2.0**	**3.0**	**3.1**	**0.85**	**0.85**(b)
DPSM 18:1-16:0	0.9		1.4			
POSM 18:1-18:1	0.7		1.1			
PNSM 18:1-24:1	0.4		0.5			
**Phosphatidylinositol (PI)**	**0.5**	**0.5**	**2.1**	**2.1**	**1.00**	**0.87**(b)
POPI	0.4		1.6			

16:0-18:1						
PAPI<br>16:0-20:4	0.1		0.5			
**Cerebroside**	**30.2**	**30.2**	**0.0**	**0.0**	**2.13**	**2.15**(c)
DBGS<br>20:1-20:0	20.5					
PNGS<br>18:1-24:1	9.7					
**Cerebroside sulfatide**	**7.9**	**7.9**	**0.0**	**0.0**	**0.61**	**0.60**(c)
PXSU<br>18:1-24:0	3.0					
PNSU<br>18:1-24:1	4.9					
**Cholesterol**	**42.6**	**42.6**	**43.8**	**43.7**	**-**	

(a) Experimental values in mol fraction derived from Inouye (1988)

(b) Derived from Manzoli (1970)

(c) Values obtained by averaging the values reported in O'Brien (1965) and Bosio (1998)
